# Robust Models for Optic Flow Coding in Natural Scenes Inspired by Insect Biology

**DOI:** 10.1371/journal.pcbi.1000555

**Published:** 2009-11-06

**Authors:** Russell S. A. Brinkworth, David C. O'Carroll

**Affiliations:** Discipline of Physiology, School of Molecular and Biomedical Science, The University of Adelaide, South Australia, Australia; Université Paris Descartes, Centre National de la Recherche Scientifique, France

## Abstract

The extraction of accurate self-motion information from the visual world is a difficult problem that has been solved very efficiently by biological organisms utilizing non-linear processing. Previous bio-inspired models for motion detection based on a correlation mechanism have been dogged by issues that arise from their sensitivity to undesired properties of the image, such as contrast, which vary widely between images. Here we present a model with multiple levels of non-linear dynamic adaptive components based directly on the known or suspected responses of neurons within the visual motion pathway of the fly brain. By testing the model under realistic high-dynamic range conditions we show that the addition of these elements makes the motion detection model robust across a large variety of images, velocities and accelerations. Furthermore the performance of the entire system is more than the incremental improvements offered by the individual components, indicating beneficial non-linear interactions between processing stages. The algorithms underlying the model can be implemented in either digital or analog hardware, including neuromorphic analog VLSI, but defy an analytical solution due to their dynamic non-linear operation. The successful application of this algorithm has applications in the development of miniature autonomous systems in defense and civilian roles, including robotics, miniature unmanned aerial vehicles and collision avoidance sensors.

## Author Summary

Building artificial vision systems that work robustly in a variety of environments has been difficult, with systems often only performing well under restricted conditions. In contrast, animal vision operates effectively under extremely variable situations. Many attempts to emulate biological vision have met with limited success, often because multiple seemingly appropriate approximations to neural coding resulted in a compromised system. We have constructed a full model for motion processing in the insect visual pathway incorporating known or suspected elements in as much detail as possible. We have found that it is only once all elements are present that the system performs robustly, with reduction or removal of elements dramatically limiting performance. The implementation of this new algorithm could provide a very useful and robust velocity estimator for artificial navigation systems.

## Introduction

The extraction of useful motion cues for navigation through visual scenes is technically challenging. While artificial systems struggle to solve this task in real time, insects with low-resolution eyes and small brains (less than a million neurons) [1] are able to avoid obstacles and successfully navigate through complex surrounds during high-speed flight [2]. This efficiency is inspiring for software engineers who struggle to achieve similar performance in artificial vision utilizing high resolution cameras, sophisticated software, and computers with hundreds of millions of transistors. Furthermore, insect vision has many unique features that lend it to useful applications. Despite inherently low resolution in even the best fly eyes [3] and visual processing that is simple and tractable enough for modeling, insects achieve spectacular flight control using passive visual sensors. Accurate models of such a system would allow replication of an insect's ability to discriminate visual scenes based on contrast, shadow, motion etc [4].

Many insects are adept at high-speed aerial maneuvers based on visual cues, using motion vision for the detection of targets [5], for visual odometry [6] and angular velocity estimation [7]. Among insects dipteran flies stand out with highly acrobatic pursuit behavior at angular velocities of several thousand degrees per second [8], although these higher speeds likely exceed the useful coding range for motion sensitive neurons [9]. Many species are also excellent hoverers, able to maintain a fixed position for extended periods of time. These extreme flight modes extend vision to the upper and lower limits of the temporal resolution described for insect higher order visual neurons [10] and make them an ideal candidate to study motion vision, in particular the accuracy of wide-field angular velocity estimation.

### Models For Motion Detection

There are four main classes of motion detection models, namely: (1) differential methods; (2) region-based matching; (3) phase-based and (4) energy-based techniques (for review see [11]). All four consist of three basic components (pre-filtering, local motion estimation and integration over the field of view) but vary markedly in the approaches used to realize these steps.

#### Differential

These methods including gradient-based models, determine velocity from spatiotemporal derivatives and models exist that employ both first [12] and second order derivatives [13]. Despite producing reasonably accurate results under a number of realistic scenarios differential methods are sensitive to the type of numerical differentiation and spatiotemporal smoothing used, as ‘raw’ methods (without sufficient smoothing) can produce discontinuous results. Due to the differentiation they are also particularly susceptible to errors under noisy conditions [14].

#### Region or feature based matching

Such techniques normally involve maximizing a cross-correlation or minimizing a difference measure such as the RMS error [15]. These also include the use of probabilistic approaches, Kalman Filters [16] and Monte Carlo localization [17], to generate and determine location on topological maps. The use of some modified neural networks to determine image velocity [18] can also be considered in this category. When accurate numerical differentiation can not be used due to noise, low frame counts or aliasing it is common for engineers to use region-based matching techniques. However these methods tend to only be accurate at high velocities and are less able to accurately estimate sub-pixel displacements. Although, unlike most other methods of velocity detection, the time required for reliable velocity estimation is generally much less and can be obtained in only 2–3 frames.

#### Phase-based

These techniques for determining image motion rely on the phase behavior of arrays of band-pass filters [19]. These filters decompose the input signal according to scale, speed and orientation. Operating in the complex domain phase-based techniques are in effect a differential technique operating on phase rather than amplitude, which has been shown to be more stable [20]. While such models have been shown to produce more accurate responses than others types of motion detection [11] they can still suffer from noise and discontinuity limitations as with gradient-based models.

#### Energy- or frequency-based

Methods that use the output energy of velocity-tuned filters to estimate motion are in this category [21],[22]. These techniques have rarely been used in practical applications as they tend to give outputs contingent on non-motion parameters of the image, can have non-trivial initial condition equations and some have underlying assumptions that are not often true (i.e. some assume the input stimulus is equivalent to white noise).

### Biological Vision Uses Correlation-Based Motion Detection

It has been shown that certain energy-based methods are equivalent to correlation-based methods [23]. Given the problems with this class of motion detection it is perhaps surprising that correlation-based models appear to be the ubiquitous form of motion detection in biology. The correlation motion detector model [24] has been used to explain direction selective motion detection in a wide variety of insects, birds and mammals, including humans [25]–[27]. This model involves a non-linear correlation of adjacent spatial samples, with an asymmetric delay filter giving rise to direction selective responses within a local elementary motion detector or EMD [24],[28]. While the term “EMD” has been used in the context of numerous variant or alternative forms of local motion detector, in insects arrays of correlation-based EMDs are then summed by so-called lobula plate tangential cells (LPTCs) to provide measurements of wide-field optical flow or motion of specific targets [29]. By analogy to insect EMDs, our subsequent use of this term thus specifically refers to EMDs based on a local correlation operation.

Two key questions arise from the observation that biological motion detectors are of the correlation class. Firstly, assuming biological vision has strong selective pressures to attain a robust and efficient system that is optimized for the task, what are the compelling advantages for this type of motion detector in the context for which they are used? Secondly, how does the biological system overcome the intrinsic problems with this type of motion detector?

### Possible Advantages of Motion Correlation

Detectors based on motion correlation have been shown to have significant advantages over gradient models [30] where detector noise is problematic [14],[31], e.g. at low contrasts or luminance. Certain features of the correlation EMD make it an extremely useful primitive for biological motion processing, particularly its robustness to both temporal and spatial noise [32]. However, such EMDs are also sensitive to non-motion-related parameters of visual stimuli, and do not by themselves give an unambiguous indication of angular velocity [33], which is at odds with the apparent ease with which insects analyze this parameter [6]. This is due in large part to the inherent sensitivity of correlation-based EMDs to contrast and spatial structure of local features within moving scenes. This leads to ambiguity in the local response as a function of angular velocity, a phenomenon we term ‘pattern noise’ [33]. However previous work [34] has suggested that static and dynamic non-linearity associated with obvious components of physiological implementation of the model helps overcome some of the inherent limitations of the basic EMD.

One contributing factor in the ability of correlation based motion models to accurately encode angular velocity is the relative consistency of the spatial statistics of natural scenes, in spite of structural difference [33]. Natural images tend to possess spatial power spectra with an approximate 1/*f* ^2+u^ characteristic, where *f* is spatial frequency and u is small (i.e. a straight line on a log-log scale) [35]. In addition to similarity between different scenes this characteristic implies a self-similarity in natural imagery at different spatial scales, although residual differences in structure remain.

A recent electrophysiological breakthrough was made showing that unlike when using sinusoidal stimuli the LPTCs of insects shown natural images robustly encoded angular velocity independently of the contrast in the scene (see Figure 3B from [36]), a characteristic not predicted by earlier models. This highlights the importance of testing biological motion detection, and models based upon it, under as ‘natural’ conditions as possible.

In this paper we provide an explanation for a controversy that has plagued visual science. How is it that biological motion detecting neurons can reliably encode angular velocity across different scenes when electrophysiological evidence shows that they use correlation-based EMDs? To do this we extend motion models, based directly on the well-studied LPTCs in the insect visual system [34],[37], by inclusion of additional dynamic non-linear components that combine to provide a robust estimate for global angular velocity and thus account for hitherto poorly understood properties of the fly LPTCs. The inclusion of these non-linearities, while overcoming many of the problems with motion energy models, is only slightly more complex computationally than the raw EMD model and far more efficient than most other motion detection algorithms. Furthermore, the model works on ‘real-world’ luminance levels, rather than the 8-bit normalized images captured by most current digital systems, making it more easily implemental on low power custom imagers.

## Methods

### High Dynamic Range Image Capture

Our primary purpose was to develop a model robust against the statistical variance between different scenes in nature, where luminance can vary by over 6 decades or more. In order to capture images for use as stimuli we therefore used a Nikon D-70 digital camera and panoramic tripod head attachment to obtain 14 panoramic images from a variety of urban and natural locations around Adelaide, South Australia in high dynamic range (HDR) format. Locations were selected to represent a range of luminance, contrast and spatial clutter conditions. Each panorama was obtained using a series of 12 overlapping panels saved in 16-bit NEF (raw) format (12-bits of actual dynamic range). Each panel was imaged at 3 different exposure levels (−2.0 and +2.0EV bracketing) in order to capture components of the scenes that exceeded the dynamic range of the camera sensor. We used PTGui (New House Internet Services BV) to stitch the 12 overlapping images together for each of the three different exposures into full 360 degree panoramas. For each panorama over-saturated pixels were discarded and local luminance was established using a linear gamma curve for the camera luminance values and cosine weightings depending on individual pixel values, i.e. low and high pixel values were assigned low weights while mid range pixels had high weights [38]. We combined the panoramas, with an offset depending on exposure, and converted them to floating point format (IEEE single precision standard) at 8000×1600 pixel resolution and full color using custom software written in LabView (National Instruments). Such high resolution was not needed for the detail, as insect optics are too coarse to make use of it, but rather to permit accurate simulation of slow image speeds. The full color HDR images are available for use by interested parties by contacting the authors.

**Figure 1:**
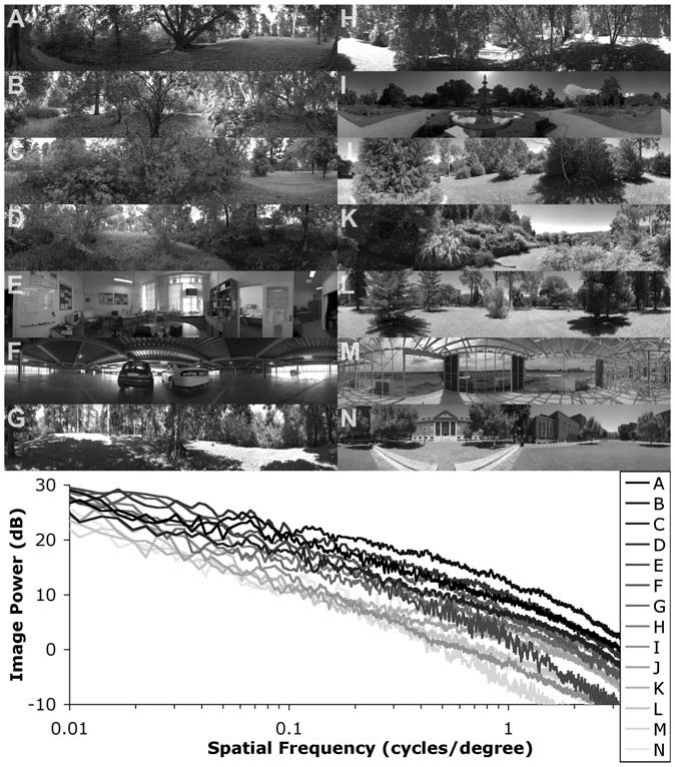
Panoramic input images. (upper) The model used high-dynamic range inputs however they have been normalized, gamma corrected and reduced to 8-bits of dynamic range for reproduction here. Images are ranked from highest to lowest contrast based on the raw elementary motion detection contrast measure (C_EMD_: see text for details) and cover a wide range of different environments and lighting conditions. Real world brightness (Cd/m^2^) and contrast values are given in Table 1. Only the green channel of the images (shown) was used as inputs to the model. (lower) Average 1D row power spectra of the 14 natural panoramic images used as inputs to the motion processing model. All have an approximately linear relationship between power and spatial frequency (on the logarithmic axis) common in natural scenes. The vertical offset (contrast) in the graphs varied almost 10dB between the different images. The roll-off at higher frequencies was caused by stitching artifacts and was outside the pass-band of the models used.

Since the motion processing pathway of insects is known to be monochromatic [39],[40] only the green channel was used as inputs to the motion detection model. All images used in this study, and the associated mean 1D row power spectra, and are shown in Figure 1. There was a larger roll-off in the higher frequency components of the images than would be expected from the non-idealities of the lens used, caused by stitching artifacts in the generation of the HDR panoramic images. The inevitable time delay between taking each of the panels resulted in small movements of the fine details in the scene (e.g. leaves) thus producing a low-pass effect. Furthermore, spatial corrections for the lens distortions and software alignment of the panels to produce panoramas may have reduced the detail in the overlapping panel sections. However, the frequency region in the pass-band of the insect LPTCs modeled in this work (<1 cycle/degree) appeared unaffected by this smoothing.

### Image Statistics

Table 1 shows the brightness and contrast for the 14 images illustrated in Figure 1. Unlike in traditional imagery HDR images vary enormously in mean luminance. In order to compensate for this, and produce contrast metrics that were not dependant on image brightness, a crude global gain control was used (divide by mean luminance). Because image normalization is a major role of the biological photoreceptors this step was omitted in subsequent modeling. Additionally, since defining image contrast is so difficult for natural scenes, we used several different measures to quantify it (Table 1), based either on the global image statistics, or taking into account the specific receptive field properties of local motion detection and the biological system it is intended to mimic [41].

**Table 1:** Image Statistics.

Image	Luminance (Cd/m^2^)	C_RMS_	C_Row_	C_Effective_	C_EMD_
A	1138	3.335	2.193	3.228	1.762
B	356	3.652	2.908	5.674	1.538
C	877	3.048	1.671	2.312	1.389
D	490	4.642	2.656	3.413	1.382
E	491	2.465	2.050	1.348	1.170
F	276	4.407	2.134	2.839	1.147
G	2715	1.455	1.666	1.686	1.072
H	1684	1.600	1.826	1.962	1.013
I	11648	0.930	1.341	0.662	0.681
J	3339	0.932	1.391	0.973	0.665
K	3901	1.140	1.448	1.277	0.644
L	5112	0.889	1.242	0.846	0.572
M	27993	0.731	1.034	0.686	0.520
N	9249	0.807	1.145	1.013	0.444
Range	101	6.352	2.813	8.565	3.972

RMS Contrast (C_RMS_) is the global standard deviation divided by global mean. As a global measure it gives a simple to calculate estimate of the contrast in the whole image and makes no assumptions about directionality. However it can produce large values simply by virtue of the fact many images contain large, yet uniform, bright (e.g. sky) and dark (e.g. ground) sections that do not necessarily produce strong local motion cues during horizontal (yaw) motion.

Row Contrast (C_Row_) is the square root of the mean 1D row power spectra. Since the neurons we were mimicking are selective for horizontal (yaw) motion having an estimate bias in this direction was appropriate. However this measurement weighted all spatial frequencies equally, a situation that resulted in more influence being given to higher spatial frequencies (fine detail) than in either the biological system or our model of it.

Effective Row Contrast (C_Effective_) is the square root of the y-intercept in the line of best fit for the mean 1D row power spectra between 0.01 and 0.5 cycles/degree (on a log-log scale) to match the observed spatial coding range for insect vision. Note that 0.5 cycles/degree is the Niquist limit for hoverfly spatial sampling, which is approximately 1 degree separation between pixels [42], while field of view of 100 degrees or more are not uncommon in fly LPTCs [43]. This measure took advantage of the linear (on a log scale) relationship between image power and frequency in natural images and also the optical limitations (spatial sampling) of the system. While this is a more insect-biased contrast measurement than either of the previous two metrics it was still essentially based on low order image statistics.

EMD Contrast (C_EMD_) is the square root of the response of a basic motion correlator model. The images were blurred and optically sampled as for motion detection (section 3.2), then passed through a basic unelaborated EMD model at a single speed, below the velocity maximum of the system. The size of the response to this raw EMD model gave an estimate of image contrast that took into account the exact conditions experienced by the motion detection model. Since the images were high dynamic range, image normalization (division by global mean) was performed so this measure of contrast was only influenced by the structure within the environment and not the absolute luminance of the image.

Comparison of the differences in contrast by these four measures confirms we achieved our objective in obtaining a set of images that should provide an enormous range in responses for a classical motion energy model tuned to similar spatial sampling. Also, while the different contrast metrics did show some differences they produced similar results, with the average correlation (r^2^) between the C_EMD_ measure and the other three approximately 0.7. Note that recent electrophysiological work using a comparable set of images (but low dynamic range) did show that neurons in the brain of the fly were able to robustly detect angular velocity independent of the scene [36].

The row contrast measurement (C_Row_) gave the smallest range of estimates for image contrast. This was due to the fact it was more heavily biased towards high spatial frequencies than the other measures and frequencies above 5–6 cycles/degree were likely to be influenced by lens distortion and stitching artifacts, hence reducing the contrast of the images. This limitation was addressed when using effective row contrast (C_Effective_) by logarithmically weighting the spatial frequency (i.e. more weight to lower frequencies) and limiting it to details larger than 0.5 cycles/degree where distortions were minimal.

### Motion Detection Model

**Figure 2:**
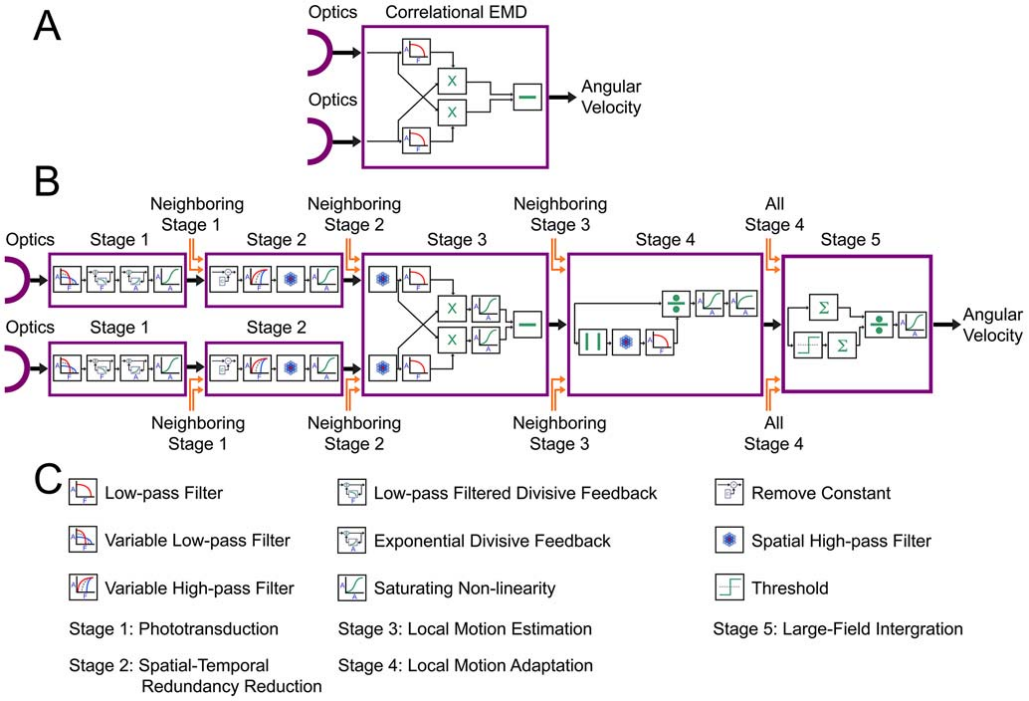
Motion processing model. A) Schematic of a basic correlator elementary motion detector (EMD) used as the fundamental motion detection algorithm in this paper. B) Diagrammatic representation of the fully elaborated motion processing model used in this study. C) Legend describing the symbolic representations used in B). Each stage of the model represents the processing occurring on a pixel-wise basis within the insect visual system. Connections between near-by processing columns (nearest or next-nearest neighbors) in the 2D network occur between stages, mostly in the form of spatial high-pass filtering, with the only global stage a final spatial summation at the start of stage 5. Each stage is further divided into smaller processing steps involving operations such as 1st order low-pass filtering, centre-surround antagonism, non-linear gains or divisive feedback. Further detail is presented in the text.

The motion detector used in this paper, shown in Figure 2, was, at its core, based on the Hassenstein-Reichardt Correlator [24]. However we added a number of elaborations (Figure 2B) to help overcome the limitations of this class of model. This more robust model took into account a number of the processing steps known, or presumed, to exist in the fly visual system and is described in the results. All stages of the model were simulated using Matlab (MathWorks).

#### Optics

The optical model used to blur and sample the panoramas was based the resolution and optical quality of the fly visual system [39],[42],[44] and pilot simulations. However, a fixed resolution and optical blur was selected rather than using settings that varied across the image as with the natural compound eye. Images were first blurred with a 2D Gaussian to simulate the optical properties of the lens (Δρ = 1.4 degrees, full width at half maximum) prior to hexagonally sampling the image as per the photoreceptor spacing in the eye (Δϕ = 1 degree, horizontal spacing between adjacent pixels).

#### EMD

Each model incorporated a basic correlational elementary motion detector (EMD) [45] with the minimum processing required to generate motion sensitive outputs. In this model a delayed version of the output from one detector is multiplied with the (non-delayed) output of an adjoining detector. The elimination of flicker and the generation of a response in the opposite direction was achieved by subtracting two mirror symmetric units. Comparisons were made between pixels centered on the current spatial location and the nearest and next-nearest neighbors. These comparisons were then weighted for directionality and position [46] before combining to produce a motion vector for horizontal motion (corresponds to yaw rotation for a panoramic image). Earlier modeling of ‘basic’ EMDs (e.g. [47]) employed first order low-pass filters with time constants in the order of 35 ms. In our model the delay element was achieved by cascading three first order low-pass filters, all with the same cut-off frequency (f_c_ = 12 Hz), and an additional fixed time delay of 2 ms. This set of parameters was chosen as it gave a biologically realistic transfer function with a small delay before a smooth rapid rising phase and a longer falling phase (approximately log-normal response). The value used produced an optimum at approximately 100 degrees/s, in line with neurobiological recordings from fly motion sensitive neurons viewing similar natural images [36], and corresponded to a temporal cut-off frequency around 5.5 Hz, similar to that found using sine wave stimuli in flies [48].

### Model Analysis

We tested the model under a range of velocities (6 points per decade) from 0.01 degrees/s to 1000 degrees/s by rotating the panoramic input images within the virtual environment. Although our modeling used discrete time we utilized a high sample rate relative to the time constants of biological vision in order to approximate continuous time processing. The sample rate of the simulation was 1 kHz for all rotation speeds below 200 degrees/s and 5 kHz for all rotations above 200 degrees/s. The working angular velocity range of the model was below 100 degrees/s, with faster rotations producing increasingly smaller responses. Thus all analysis was limited to the range 0.1 degrees/s to 100 degrees/s. We employed linear sub-pixel interpolation during the simulated yaw rotations to ensure an accurate simulation of smooth motion at low velocities.

In order to avoid ‘neural after-images’ the initial conditions were set to the mean luminance of the image. Simulations were run for 1050 ms to allow sufficient time for the system to reach steady state. All analysis was based on the average response of the last 50ms.

Two parameters were calculated to quantify the output of the model at each angular velocity in terms of image invariance.

Coefficient of Variation (CV) was defined as the standard deviation of the response of all images at a given rotational speed divided by the mean of the responses and is shown in equation 1. This parameter was used to show variation (ambiguity) in model responses to different images at a specific angular velocity. Lower coefficients of variation meant less variability and a more reproducible result across different images. However, having a low CV does not automatically make a system a good velocity discriminator. Overlapping horizontal lines will have a low CV but will produce the same output value for a range of velocities, making it impossible to distinguish between different image speeds.
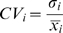
(1)Where CV*_i_* is the coefficient of variation at point *i*, σ is the standard deviation of the image responses, 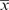 is the mean of the model responses to the images and *i* is the test velocity. CV is expressed as a percentage in the text.

Z Score was defined as the difference in the means at the two consecutive velocities divided by the sum of the two consecutive standard deviations then scaled for the number of samples per decade (i.e. local slope divided by local variability) and is shown in equation 2. Unlike CV this parameter represents the ability of the system to discriminate between velocities. A higher Z score meant that the ability to determine the difference between velocities was greater.
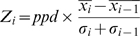
(2)Where Z*_i_* is the Z score at point *i*, *ppd* is the number of test points per decade (in this case 6), 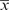 is the mean of the model responses to the images, 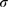 is the standard deviation of the image responses, *i* is the test velocity and *i*−1 is the previous test velocity.

All results are given in the form mean±95% confidence interval unless otherwise stated. Global CV or Z score statistics were calculated as the average over the range 0.1 to 100 degrees/s. This range was chosen as the maximum closely matches the optimal point seen in biological motion detecting neurons [36] and the minimum is within the accuracy of the animation method used to simulate image motion (linear interpolation). However the model parameters could be altered to create a different coding range if desired.

## Results/Discussion

Being one of the most extensively studied systems in neurobiology the fly motion system [7],[45] was used as the base line for all variables (such as time constants, gain factors etc) in the model where available. Where such data did not exist, or was ambiguous, a best estimate was used that was consistent with typical values found in other neuronal systems. In such cases a small amount of parameter optimization was used to ensure accurate coding was not compromised. It was not unusual to find a parameter could take a range of values without having a significant impact on angular velocity coding, i.e. the system was not critically dependant on the exact values used.

### Motion Detection Model

**Figure 3:**
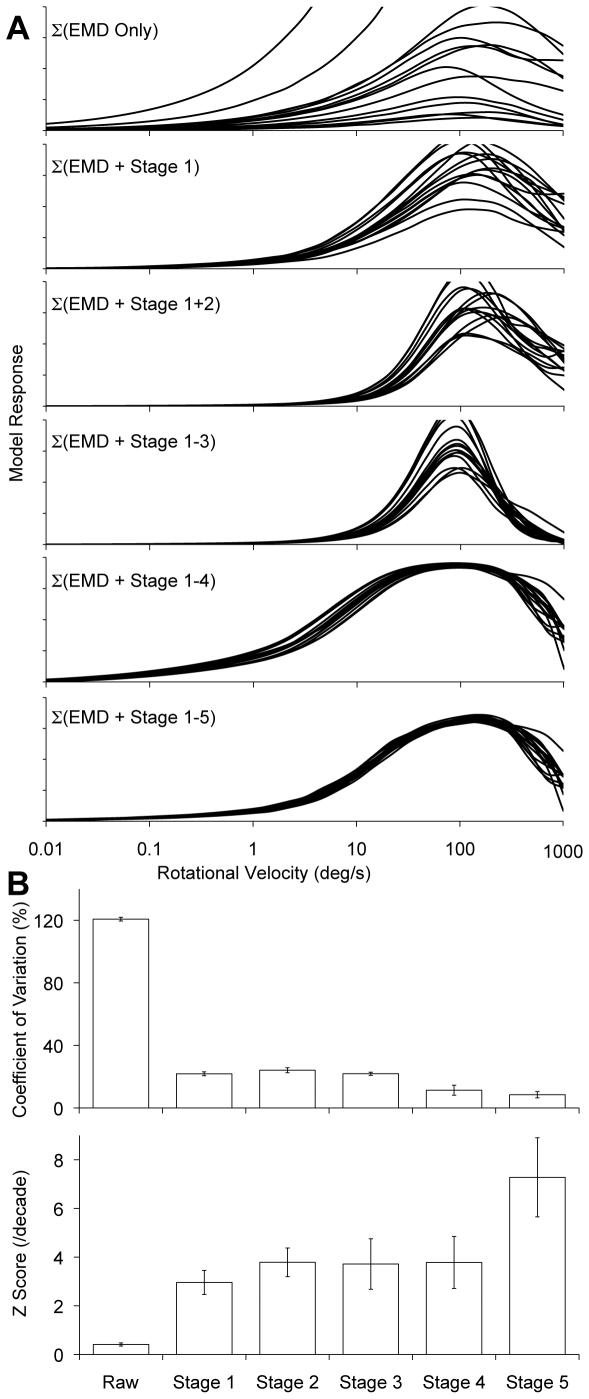
Model responses after various processing stages. A) Steady-state responses, integrated over the entire image, of the model to all 14 input images over the range of velocities tested after inclusion of various modeling stages as depicted in Figure 2. Lines are color coded to the images as shown in Figure 1. B) Summary statistics of model performance after each stage of processing. All data are given as mean of responses over the range 0.1–100 degrees/s. Error bars represent 95% confidence intervals. The inclusion of each of the stages improved the ability of the model to reliably encode velocity by reducing the variability in the response between images. Σ illustrated that the responses were summed over all space, EMD stands for correlational elementary motion detector and was the fundamental motion estimation operation. For a detailed description of the processing of each stage, and the exact effects on velocity consistency between images, see main text.

Each stage of the model depicted in Figure 2B was built up sequentially in order to investigate the contribution of each stage to reliable angular velocity encoding. The response of the model to each of the 14 images, and the effect of adding each of the processing stages into the chain, is shown in Figure 3. As with all correlation-based EMD models the system produced ambiguous responses, with the same signal value for two different velocities either side of an optimum. However in practice this limitation could be overcome by using the system only within the coding range (i.e. below the optimum).

#### Basic EMD

Consistent with Dror et al [33] the basic EMD model (Raw) gave broadly similar shaped angular velocity tuning curves over the range tested and peaking at around 100 degrees/s, but with huge variance in the response gain as a function of angular velocity (CV = 121±1.17% and Z score = 0.408±0.063 in the range 0.1 to 100 degrees/s). Hence making it completely unusable as an angular velocity estimator as the response at any one angular velocity was vastly different for each image.

#### Stage 1 – phototransduction

This stage was a model to account for the non-linearities in blowfly phototransduction, and was based on our modified version [49] of a parametric model initially proposed by van Hateren and Snippe [50]. This included dynamic pixel-wise control of several parameters: gain, the corner frequency of a low pass temporal filter, dynamic gamma correction and a saturating non-linearity (Naka- Rushton transform). These all resulted in a useful dynamic range compression by increasing the gain of dark sections of the image while simultaneously and independently reducing the gain in higher luminance sections. This processing has been shown to be functionally equivalent to that found in primate cone receptors [51] and also facilitates the detection of small targets in clutter [52]. All parameters were set to those found in our previous photoreceptor recordings [49].

The inclusion of the biomimetic photoreceptor processing improved the performance of the model by over 600% compared to that from the unelaborated (raw) EMD model. However the performance of the system as a reliable angular velocity estimator was still quite low. Coefficient of variation (21.9±1.22%) and Z score (2.96±0.495) values showed the variation between scenes still represented a significant portion of the entire response. The addition of this stage moved the model from what has been previously only attempted using normalized low dynamic range images [34] into a form that could be used under real-world luminance inputs with no pre-conditioning.

#### Stage 2 – spatial-temporal redundancy reduction

This stage was designed to account for additional processing by the second-order neurons, lamina monopolar cells (LMCs) in flies, which are analogous to bipolar cells in mammalian eyes [53]. They remove redundancy in both space and time in an information theoretic optimal way based on the local light level [54]. Processing steps included variable (higher cut-off in areas of higher luminance) and relaxed first-order high-pass filtering (permitting some DC component of the signal to be propagated) in both space and time depending on light levels [55] and a saturating non-linearity (tanh; see equation 3). The sign inversion seen in neurophysiological recordings from these cells was not included as it had no impact on the performance of the system [56]. Similarly neural superposition was not included as it would have served no purpose. Neural superposition involves the combination of a number of (in the case of the hover-fly 6) independent samples of the same point in space to reduce noise [57]. Since this simulation had essentially no detector or model noise (or none that would be influenced by this) it was excluded.

In the case of implementing spatial high-pass filtering the ‘surround’ was defined as the response from the neighboring 6 pixels on the hexagonal grid. These signals were inverted, attenuated, delayed and smoothed (proposed operation of the amacrine cells in the biological system) before combining with the signal from the centre pixel [58]. Where possible the changes in filtering due to luminance conditions were based on previously published recordings from fly LMCs [59].
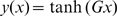
(3)Where y(x) is the output signal limited to the range ±1 (designed to mimic the limited bandwidth in a physical system) and G is the input gain.

The inclusion of LMC-like processing after the addition of stage 1 to the raw EMD model produced minor but mixed results. There was a non-significant 28% increase in the average Z score, meaning the ability to distinguish between velocities was slightly improved. However it also caused a 10% (not significant) increase in CV, resulting in slightly more variability in the responses produced by the difference scenes. Thus the LMC processing provided little extra benefit in this configuration. This is itself was surprising since the processing of the LMC (spatial-temporal high-pass filtering) has been considered an important element in the pre-processing of motion detectors based on EMDs [33]. However, this model for LMC processing does not fully capture all the non-linear components of LMC operation and it is likely missing sections play an important role in this, or other, visual tasks.

While it is clear that photoreceptors must be on the motion processing pathway there remains debate about subsequent neuronal stages with proponents both for [60],[61] and against [62] the inclusion of LMCs on the motion pathway. We decided to keep the LMCs in the model as the processing (high-pass spatial-temporal filtering) was theoretically beneficial to both motion processing and optimizing information transmission in limited bandwidths. However, we also performed tests with this stage removed to determine the actual effect the LMC model had on the reliability of angular velocity coding (below).

#### Stage 3 – local motion estimation

This stage incorporated hypothetical elaborations to the core EMD. These elaborations were additional stronger spatial high-pass filtering (nearest and next-nearest neighbors on the 2D image plane), for which there is some anatomical evidence [63], as well as additional saturating non-linearities (as per equation 3) after the multiplication (correlation) between the delayed and undelayed spatially separated pixels. The basis for this saturation was that biological neurons have a limited bandwidth, so expansive non-linearities (such as multiplication) must be bounded. Soft saturation, such as that produced by a tanh function, is commonly seen in biological sensory systems and has been proposed by others to exist in the motion pathway [64] in order to account for certain contrast tuning properties of LPTCs [65]. Unfortunately recordings from the insect medulla region, the second optic lobe neuropil and the region believed to contain the EMD-like processing, are difficult and rare [66],[67] due to the relative difficulty in obtaining stable recordings [68], so the gain was estimated to provide a good compromise between utilizing the available bandwidth and producing saturating responses.

The inclusion of the saturating non-linearities and further spatial high-pass filtering had little beneficial effect on either the average CV (9% reduction) or Z score (2% reduction) within the operating range (<100 degrees/s). However it did increase the rate of roll-off and the similarity between images at high speeds (Figure 3A). Damping in this section of the velocity curve has been shown to be important from a control systems point of view in reducing potential instability in the system during periods of very high rotational velocities [69].

#### Stage 4 – local motion adaptation

This stage was a novel model for local motion-dependent gain reduction (local motion adaptation) as observed in the rotational motion sensitive neurons in the fly visual system [70]. The motion adaptation was implemented via divisive feedback of a spatial-temporal low-pass filtered version of the local motion signal (nearest and next-nearest neighbors on the 2D image plane). This feed-forward gain control was made direction independent, as shown in biology [71], by the addition of a full-wave rectifier on the input to the low-pass filters. This motion gain control permitted a form of predictive coding where the gain in regions of high motion-energy (clutter) was reduced and the signal amplified in regions of low clutter. The basic premise was to increase the statistical independence of local motion signals by reducing their co-variance, hence increasing the information content in the global signal. Unlike the motion control used in our previous modeling [34] this new type of adaptation did not act as a contrast normalization stage. Hence the model retained one of the most curious recent findings in the fly that can not be accounted for by the work of Shoemaker et al. While images of different contrast produce similar outputs in LPTCs artificially reducing the contrast of images results in a reduction of the responses, but the responses are still similar across the different images [36]. Other processing stages included were a static saturating non-linearity (tanh; 2^nd^ last block in stage 4 of Figure 2B) and a compressive non-linearity shown in equation 4 (last block in stage 4 of Figure 2B).
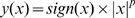
(4)Where *y(x)* is the compressed output of the local motion gain control stage, *x* is the local motion estimation after the local gain control and saturating non-linearity and *p* is the power used to compress the response range (i.e. boost the response to low speed rotation relative to that of high speed rotation). The nominal value for *p* was 0.5. This value was chosen in order to partially correct for the square-like expansion caused by the multiplication in the EMD stage and to produced a signal that was log-linear over most of the signaling range, another unexpected neurophysiological finding by Straw et al [36]. Since *p*<1 it was necessary to use the modulus of the local motion signal to produce real results. The directionality of the result was maintained by the use of the *sign* function that produced −1 if *x*<0 and 1 if *x*>0.

This processing had little effect on the average Z score (2% increase), but did reduce the average CV (48% reduction) and decrease the required output bandwidth by boosting the response to low velocities while suppressing high velocities. So while in a noise free simulation, such as that presented here, there was no real improvement in the ability to accurately encode velocities this stage will have implication in real-world implementations where noise and limited bandwidth are important considerations. It is also important to note that in previous modeling the inclusion of ‘local motion gain control’, either on its own or in tandem with other processing, constantly made angular velocity coding worse [34].

#### Stage 5 – large-field integration

This stage was a representation of the processing performed by the LPTCs in flies and was the summation of all stage 4 outputs and represented the first, and only, global calculation in the model. The low number of global calculations means that it is easier to construct physical models, such as aVLSI [34],[72] or FPGA implementations. Note that this is the first stage for which there is evidence of global integration in the biological system. This stage included a non-linear spatial correction factor [73] designed to account for the fact that not all EMDs will be activated by a given natural scene at any instant. This was followed by a final saturating non-linearity (tanh; equation 3). This stage did not contain additional global components of motion adaptation such as the famous ‘waterfall effect’, which are a known feature of biological visual systems [74]. In the absence of a clear role for these phenomena in velocity coding they may add an unnecessary level of complexity to artificial systems required to estimate actual rather than relative angular velocity.

Adding this final stage to the rest of the processing chain had a marked positive improvement on both the CV (25% reduction) and the Z score (93% increase), making the model much more robust. However, it should be noted that the improvement of the model response after the inclusion of stage 5 was not solely due to the performance of that stage but rather the accumulated actions of each of the preceding stages. The replacement of the photoreceptor model for a standard normalization operation (divide by image mean), while still maintaining all other operations, reduced the average Z score from 7.28±1.62 (mean±95% confidence interval) to 1.42±0.42. The removal of stage 2 from the complete model caused the average Z score to drop by over 40% to 4.35±0.77, despite this stage having had no significant effect when added after stage 1 in the absence of other elaborations. These findings highlight the importance of looking at the performance of the system as a whole rather than the individual components of the model.

### Effect of Optical Sampling

The response of the modeling showed that the inclusion of bioinspired processing components could, in tandem, produce reliable angular velocity coding of visual inputs. However it was important to determine the requirements of this approach from an optical sampling view-point. In order to test the robustness of angular velocity coding for different spatial sample rates, we ran the full model for a range of possible constant sampling optical configurations. The spatial baseline used was the hoverfly (*Eristalis tenax*), where resolution (Δϕ) is maximally about 1 degree but can drop off to almost 2 degrees in the periphery [42]. Other types of flies can have even less resolution, e.g. Land [75] reports 2.8 degree resolution in house flies (*Musca domestica*) and as low as 5.8 degrees in fruit flies (*Drosophila melanogaster*). Furthermore acceptance angles (Δρ), which can be approximated by a Gaussian blur with a full width at half maximum of 1.4 degrees (standard deviation of 0.59 degrees) in hoverflies [44], can be as large as 2.6 degrees in bees [76] and even 4 degrees in dark adapted locusts [77].

**Figure 4:**
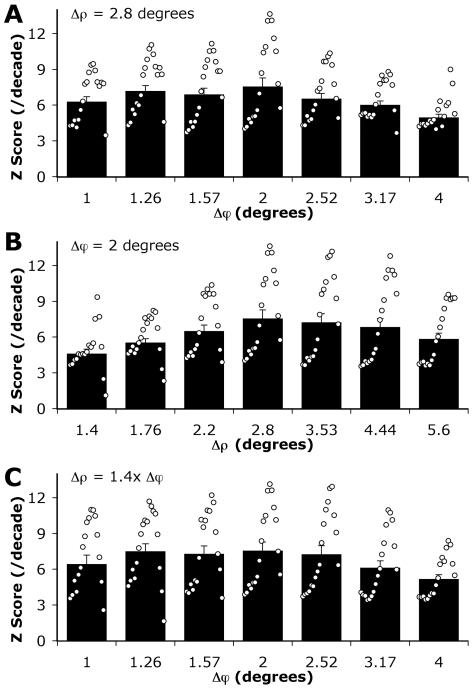
Effect of image blur and spatial sampling on velocity discrimination. A) the optical blur (Δρ) was a constant 2D Gaussian of 2.8 degrees (full width at half maximum) and the spatial sampling rate (Δϕ) was varied. B) The spatial sampling was set to 2 degrees (180 pixels in horizontal dimension) and the optical blur varied. C) The optical blur was fixed at 1.4 times larger than the spatial sampling rate, which was varied. The circles show, in order, the Z score between each of the 19 tested speeds between 0.1 and 100degrees/s inclusive (equally spaced on a log scale, i.e. 18 intervals). The columns and error bars show mean Z scores and standard errors of the mean respectively. In each case it was found that the maximal Z score (i.e. best discrimination between velocities) was with a spatial sampling of 2 degrees and an optical blur of 2.8 degrees.

The results of varying Δρ and Δϕ are shown in Figure 4 and at no time did we attempt to mimic the variable resolution found to exist across the biological compound eye. In all cases the optimum condition (producing the largest average Z score) was a Δϕ of 2 degrees with a Δρ of 2.8 degrees. When Δρ was kept constant at 2.8 degrees (Figure 4a) all tested values of Δϕ resulted in significantly lower Z scores than the case of 2 degree sampling, except for 1.26 degrees (p<0.05). However this solution came at the expense of increased computational effort, with 2.5 times more samples (and hence processing power) required to realize it.

At a fixed Δϕ the location of the optimal angular velocity (corresponding to the largest Z scores) can be shifted to higher velocities by increasing Δρ. In this system it was found that there was no significant difference in the reliability of angular velocity coding when using Δρ of 2.8, 3.53 and 4.44 degrees and with no difference in the number of calculations required to produce these results (assuming the blur was not performed by software convolution of an over sampled system, in which case smaller blur would be less computationally expensive) then the selection of blur would depend only on the application, with systems with larger blurs tuned for higher velocities.

By keeping the Δρ/Δϕ equal to 1.4 (Figure 4c) it was possible to show that the system performance was not significantly different over a range of spatial sampling values (1.26–2.52 degrees). As with the constant sampling case increasing the absolute blur moved the optimal point to higher velocities. However in our model computational time increased between these limits by a factor of 4. In computation, as in biology, greater efficiency might thus make lower spatial sampling rates more desirable.

Overall the optical model found to produce the most accurate angular velocity coding was achieved using a Δϕ of 2 degrees and a Δρ of 2.8 degrees. While the spatial sampling rate is lower than that found in the majority of insects the blur to sampling rate ratio (Δρ/Δϕ ) of 1.4 is the same as that seen in bees [76],[78] and flies [42],[44]; a ratio that has been predicted as optimal in an information-theoretical sense [79],[80]. In comparison experiments in primates have shown that the detection of high temporal frequency stimuli is governed by the relatively low resolution magnocellular pathway [81]. Furthermore, throughout the animal kingdom, ranging from invertebrates to vertebrates including humans, the mechanisms underlying motion detection can be attributed to correlational EMD-like processing [82]. Thus there is substantial evidence for a common strategy of low-resolution motion vision in many biological systems.

The reason that the optimum spatial sampling rate is so low is because the system was tested under both natural and urban images. Natural scenes have a fractal pattern (self-similarity at different scales) that means, in general, more information can be gained by increasing the resolution of the image. In contrast urban scenes (such as indoor locations) have a high degree of spatial redundancy (such as uniformly painted walls), where increasing the resolution provides little increase to the overall information gained. Since the EMD is a motion energy model it relies on information change between pixels, if there is little information change there is little energy and hence a small motion signal. Thus increasing the resolution had little or no effect on the velocity consistency of the natural scenes, as they all tended to scale together, but it did cause the urban scenes to produce relatively smaller responses. Hence the ideal spatial resolution of a system may be dependant on the mix of urban and natural environments it needs to operate in. This finding is in direct opposition to the current trends in cameras and computer vision towards support for systems with higher spatial resolution.

Unlike in most traditional artificial systems the optimum condition for this system was not a sharply focused image. This is because, due to the low spatial resolution, the system needed to detect sub-pixel motion in order to reliably encode slow velocities. If there were no optical overlap between pixels this would not be possible. However with overlap it was possible to detect small motion changes both within a given pixel and also in the neighboring pixels. Conversely, too much optical blur made the differences between the pixels too small, hence reducing the independence of each sample and resulting in less accurate angular velocity detection.

### Varying Model Parameters

**Figure 5:**
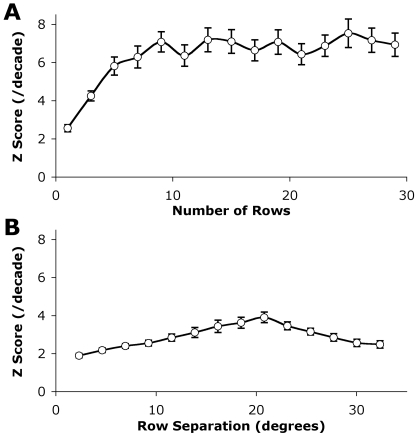
Effect of varying the vertical rows in global summation stage on velocity discrimination. A) The number of rows used in the global summation (stage 5 in Figure 2) was varied. In each case a row centered on the horizon was used and other rows were equally separated both above and below the horizon. Little improvement is gained by averaging more than 7 rows together. B) Only 3 rows were used in the calculation but the separation of the rows from the centre was varied. The optimal separation of rows occurred at approximately 21 degrees, i.e. rows at +21, 0 and −21 degrees with reference to the horizon. Due to the large amount of image similarity in the vertical dimension separations less than this resulted in samples that were not distinct enough to assist with velocity estimation. Values were calculated as the mean Z score across all velocity intervals used, error bars represent one standard error of the mean.

Although it is possible to elicit a motion response by stimulating only two adjacent receptors (see Figure 2A) integration over a larger area reduces phase dependant pattern noise [33]. The optimum integration size will be task-dependent. For the special case simulated in this paper of ‘pure yaw’ (e.g. as needs to be compensated for by a hovering fly) complete elimination of pattern noise in the time domain can be achieved by sampling across the full 360 degrees of the horizontal visual field. However, what additional spatial summation is required to reduce variability due to differences in spatiotemporal contrast over the vertical extent of the field of view? To address this, we varied the number of vertical rows averaged in stage 5 to investigate the degree to which spatial integration across a larger receptive field influenced angular velocity coding. In this case, we used the fully elaborated model (i.e. all stages), with 2 degree spatial sampling and 2.8 degree optical blur as previous experiments had suggested this to be an optimum optical design due to its compromise between Z score and computational efficiency (section 3.2). All conditions involved a central row around the horizon and an equal number of rows evenly spaced above and below the centre up to a maximum of 72 degrees (29 rows due to hexagonal sampling and the inability to use the outer most rows). The results are shown in Figure 5. The maximal average Z score was obtained by using 25 rows, however due to the logarithmic shape of the curve using any number of rows greater than 7 produced results within 10% of the full resolution.

**Figure 6:**
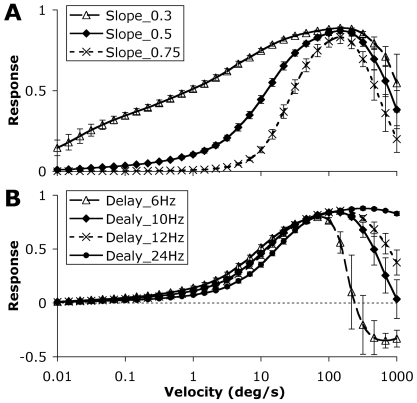
Effect of varying the working range and velocity optima. Average model responses for all 14 natural images. The model can be configured to detect different velocity ranges and to have a peak response at different velocities. Error bars represent one standard deviation. A) The slope refers to the exponent of a power function implemented at the end of stage 4. Despite the clear reduction in response amplitude at the higher slope value there was no significant difference in Z score between a slope of 0.3 and 0.75 in the range 0.01–1 degrees/s (paired t-test, p>0.05). This finding is most likely due to the lack of noise in the model and would change in any real-world implementation. B) The peak response of the model is a function of the (3rd order) delay filter implemented in the Hassenstein-Reichardt detector (stage 3). Using a slower delay filter shifts the maximum response point to lower velocities and increases the rate of roll-off outside the pass-band. In the case of the slowest delay filter used the response is inverted (aliased) at very high velocities.

The effect of varying the slope of the curve and point of maximal response is shown in Figure 6. The slope of the model can be modified to fit the desired scope of velocities for a given application. Using a smaller range (i.e. greater response gain as a function of angular velocity) made the system more robust against noise, which is more likely to be a problem at low speeds or where the output bandwidth is limited. However in a noise free simulation there was little or no benefit in reducing the working range. The average Z score was 6.77±1.16, 7.53±1.62 and 7.15±1.59 (mean±95% confidence interval) for slope parameters of 0.3, 0.5 and 0.75 respectively.

The maximum (optimal) angular velocity of the system could be changed depending on the requirements of the system (Figure 6b). In all cases the variation between images was much greater outside the working range (above the optimum angular velocity). This is because the variability within individual images (pattern noise) increased with angular velocity and with a decreasing response to the true angular velocity the signal became swamped with noise.

### Dynamic Stimuli

**Figure 7:**
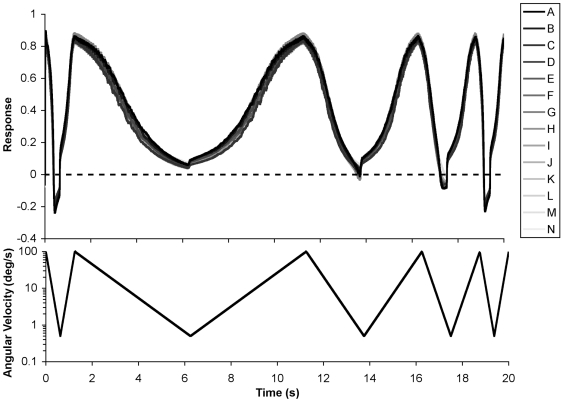
Response of the motion detection model to dynamic stimuli. The time domain response of the model to all images as tested under velocity ramps of different slopes. Image assignments and line coloring is the same as Figure 1. Despite the relatively large, and variable, accelerations involved the motion model produces very consistent responses for the different images. Aliasing (reversal of signaled direction) can be seen when the stimulus changes from decreasing to increasing velocity under the high acceleration.

In all of the test conditions described in the paper to date the image angular velocity was constant and the motion detection model was given sufficient time to reach steady-state before the results were taken. However this is not a realistic situation for a motion sensor that would typically be required to produce a reliable response under dynamic conditions. In order to test the model under conditions of variable angular velocity and acceleration a 20 second stimulus was constructed that consisted of variable width periods of constantly (in the log domain) increasing and decreasing angular velocity. The exact waveform, and the model response, is shown in Figure 7. With the median of the coefficient of variation being 7.28% the model showed little variation in response to the different scenes, even under rapid accelerations (±844 degrees/s^2^). In fact the median coefficient of variation under constant conditions over the same rotational velocities (100−0.5 degrees/s) was 6.91%, indicating a decrease in performance reliability of less than 5.5% under dynamic conditions where the model was not permitted to reach stead-state.

Despite the system only being tested under positive angular velocities there were situations when the model produced negative results indicating that the model got the direction of motion wrong. This aliasing occurred at low velocities following high velocities and at the point where the stimulus went from decreasing to increasing rotational velocities. Moreover, it was more prevalent under larger accelerations. Despite not being explicitly included in the motion model (see section 3.1 stage 5) this result is somewhat analogous to the waterfall effect where after the rapid removal of a large motion stimulus motion detecting neurons tend to hyperpolarize.

Although flies are capable of extreme angular accelerations during saccades, much larger than the 844 degrees/s^2^ tested here, [83] it is not clear that the visual system is used for coding under such situations. Some authors (e.g. [69]) have made the point that the visual motion response may be deliberately damped to avoid sensitivity to such events in order to avoid instability in the optomotor response, in lieu of a mechanism for saccadic suppression (as in primate vision) otherwise required. Other sensory systems likely play a role in encoding high-speed acceleration (e.g. halteres) and the visual motion pathway seems deliberately tuned to low speeds in flies (see [84]).

### Horizontal Field of View

In all previously described results the full 360 degrees of horizontal visual space was integrated in order to remove the dependence of the result on the part of the image being analyzed (pattern noise). Although it can be reduced by integrating over smaller areas [85] using a fully panoramic field of view has been show to be the only way to eliminate the periodic responses dependant on image statistics [86]. Behavioral experiments in the fly have shown that they are sensitive to the contrast and orientation of patterns at the level of individual receptor pairs (i.e. single EMDs) [87] or when they cover a larger (non-panoramic) area of space [88]. While not realistic for the output of a single neuron [89] the outputs of populations of motion sensitive LPTCs combine to give an almost complete panoramic view, as evidenced by the output of neck motor neurons [90], thus minimising pattern noise by means of spatial integration [87]. In addition to integrating over wider fields of view incorporating saturation and other non-linear processing elements predicted to exist in the biological motion processing pathway can modify and reduce pattern noise when a limited field of view is used [33],[91].

**Figure 8:**
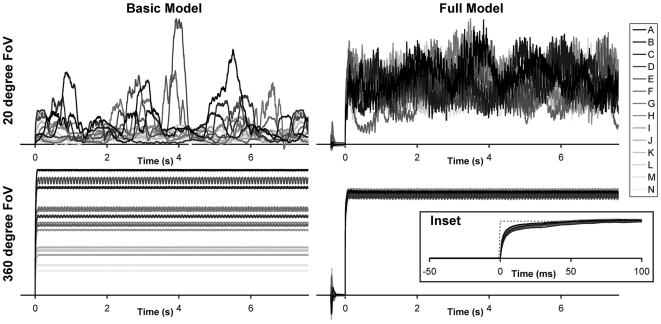
Temporal response of spatially restricted basic and elaborated models. Image appeared over a content grey background at time −400ms. Due to adaptive elements in the early visual processing an appearance artifact is visible in the elaborated models. Images were kept stationary until time 0 when the velocity stepped up to 50 degrees/s. In addition to the raw correlator elementary motion detector (Figure 2A) the basic model also included a normalization factor for the image brightness. The vertical scales for the model settings are different in each case in order to show the full variations under each condition. The variation in the time domain response (pattern noise) when the model had only a 20 degree field of view (FoV) was much larger than when averaged over the full 360 degrees of visual space. Furthermore, the inclusion of the model elaborations reduced not only the variations between images but also the pattern noise within individual images, even in the absence of a large field of view. Inset shows a close-up of the model response to the velocity step (shown as dotted gray line). Even at the very high acceleration induced by this stimulus the model maintains a similar response profile for all images tested. Image assignments and line coloring is the same as Figure 1.

In order to investigate the role of horizontal field of view on the temporal response of the system we reduced the field of view of the model to 20 degrees of visual space in both the raw (but normalized for image brightness) and fully elaborated models. Under such conditions there were two important sources of variability to consider, that between images (inter-image, as already reported) and that within images (intra-image, i.e. the time domain variability of the system in response to a constant input). The responses of the model to a constant velocity of 50 degree/s are shown in Figure 8. With the limited spatial integration and most basic EMD model the average coefficient of variation within images over a full rotation was 66.3±27.1% (mean±standard deviation) and the variation between the 14 image means was 46.6%. Thus showing the response was not a constant indicator of individual image velocity, or a good inter-image velocity estimator. Increasing the field of view to 360 degrees dramatically reduced the intra-image variation to 0.82±0.44%, however as expected it had no effect on the intra-image variation. Using the full model with the limited field of view resulted in an intra-image variation of 23.9±5.1%, reduced compared to the case with the spatially limited raw model but not to the same extent as with the panoramic view. The inter-image variation in this case was 11.7%, much improved over both raw cases. Finally, the full model with full 360 degree field of view produced both the smallest intra-image (0.48±0.14%) and inter-image (2.2%) variations. The difference in the inter-image variations between the full and limited field of view tests was due to both the reduced saturation (in the spatially limited case) and the different weighting factors in the non-linear global summation stage.

### Conclusion

By constructing a model for motion detection based on elements known, or suspected, to be present in the biological system we have shown that accurate and robust detection of global motion can be achieved using a system with very low resolution based on relatively simple mathematical operations. The key to the operation of the model was the way multiple non-linear elements interacted to produce an estimate of angular velocity that was independent of the scene it was viewing. Moreover, the performance of the system as a whole was greater than the linear addition of the individual components taken in isolation.

While we have based our model on parameters derived from physiological analysis of the fly motion pathway the model may also be applicable to data from other species. In previous work Ibbotson described ‘velocity tuned’ (VT) neurons in the honeybee that appear to differ from our model and fly neurons in having monotonic responses to very high speeds (1000 degrees/s) and apparently less dependent on spatial period of square-wave patterns [92]. While a degree of pattern invariance may result from the adaptive nature of our model, the apparent lack of response roll-off in the Ibbotson data is more difficult to reconcile with the fly data. Interestingly, however, because the bee spatial optimum is much lower than in flies (coarser spatial sampling) and the temporal optima much higher (shorter delay) [84], the useful “coding range” (as referred to in our model description) is predicted to be shifted to 10 times that in flies (see [93]), where velocity optima for natural scenes are already 200 degrees/s [36]. Since the Ibbotson data set only explored velocities below 1000 degrees/s it is thus likely that patterns were not animated at high enough velocities to see the response roll-off predicted by a correlation-based model (including our fully elaborated model).

There is strong evidence that the fly motion pathway processes negative and positive contrasts separately [47]. However, the motion model described here does not incorporate any kind of ‘contrast asymmetry’. Although several authors have explored whether the motion pathway is fed by separate ‘on’ and ‘off’ pathways (e.g. [94],[95]), no studies have yet provided conclusive results. Recently we have shown that the separation between ‘on’ and ‘off’ pathways can be a useful primitive in target detection [96],[97]. While others have shown that contrast separation can be used as a pre-processing stage in a different type of EMD-based model [98] the current model shows it is not a necessity for the accurate detection of wide field angular velocity using correlation-based EMDs.

There is a significant push to reduce the complexity of bio-inspired algorithms so they will run in real-time on modern computer platforms [99]. The complexity of the model described in this paper may be too much to realize in a real-time application based on a single serial CPU. However its highly parallel nature and low resolution make it an ideal candidate for implementation in either a FPGA or GPGPU [100] based platform. Furthermore reduced versions have already been produced in analog VLSI [101] and may be suitable for serial digital systems as well [102] where frame rates in excess of 100Hz have already been achieved using standard consumer-level computers. It is also important to note that the computational complexity of an EMD based system can be orders of magnitude less than alternative schemes for computing local velocity vectors in optic flow analysis (e.g. [103]).
